# Efficient Separation and Targeted Activation of Lignin by Ethanolamine Pyruvate Protic Ionic Liquid

**DOI:** 10.3390/polym18091109

**Published:** 2026-04-30

**Authors:** Liuli Zhu, Jiatian Zhu, Jingpeng Zhou, Qin Feng, Baojie Liu, Chengrong Qin, Chen Liang, Caoxing Huang, Shuangquan Yao

**Affiliations:** 1 Guangxi Key Laboratory of Clean Pulp & Papermaking and Pollution Control, School of Light Industrial and Food Engineering, Guangxi University, Nanning 530004, China; 2 Shandong Huatai Paper Co., Ltd., Dongying & Shandong Key Laboratory of Biobased Material and Green Pulp Papermaking, Dongying 257335, China; 3 Jiangsu Co-Innovation Center of Efficient Processing and Utilization of Forest Resources, Nanjing Forestry University, Nanjing 210037, China

**Keywords:** lignin, protic ionic liquid, phenolic hydroxyl, depolymerization, biomass fractionation

## Abstract

To address the challenges of inefficient depolymerization and undesirable condensation side reactions of lignin in lignocellulosic biomass, this study employed an ethanolamine pyruvate protic ionic liquid (EAP) pretreatment system to achieve selective separation of lignin from eucalyptus while simultaneously enabling its in situ structural activation. Under optimized conditions (pyruvate-to-ethanolamine mole ratio of 1:3, 120 °C, 40 min), the EAP system afforded a lignin separation yield of 79.0 ± 0.6%, with dissolution yields of cellulose and hemicellulose of 9.6 ± 0.3% and 11.2 ± 0.4%, respectively. According to 2D-HSQC NMR and ^31^P NMR analyses, the relative content of β-O-4 ether linkages in the isolated lignin decreased from 18.4 ± 0.4% to 14.2 ± 0.3% after EAP treatment. The total phenolic hydroxyl content reached 2.26 ± 0.08 mmol/g, and the syringyl-to-guaiacyl (S/G) ratio declined from 1.72 ± 0.04 to 0.71 ± 0.03. Based on these observations, it is proposed that the ethanolamine component facilitates the dissociation of the lignin network through hydrogen bonding and stabilizes reactive intermediates, while the pyruvate component participates in the cleavage of β-O-4 ether linkages and the removal of methoxy groups via proton catalysis and nucleophilic attack. Compared with the ethanolamine and ethanolamine acetate systems, EAP pretreatment yielded lignin of higher purity (98.4 ± 0.3%) under milder conditions, and the isolated lignin exhibited stronger antioxidant activity (IC_50_ = 0.17 ± 0.02 mg/mL). This work offers insights into the development of pretreatment systems that combine efficient separation with structural preservation of lignin.

## 1. Introduction

Lignocellulosic biomass is renewable and features a zero-carbon cycle. It serves as a key substitute for fossil resources in achieving carbon neutrality and plays an irreplaceable ecological role in developing a green, low-carbon circular economy [1,2]. Lignin, a natural carbon reservoir within aromatic polymers, possesses abundant phenylpropane units in its three-dimensional network. This structure confers a unique advantage for producing high-value chemicals and functional materials [3,4]. However, the dense, cross-linked network formed between lignin and polysaccharides in plant cell walls, together with the irreversible condensation reactions that readily occur during conventional pretreatment, pose significant challenges to the efficient and selective depolymerization of lignin [5,6].

Ionic liquids (ILs) have gained considerable attention in lignocellulosic biomass pretreatment due to their tunable cation–anion structures, good lignin solubility, and relatively mild reaction conditions [7,8]. Rational design of the cation and anion enables selective lignin separation while minimizing the degradation of carbohydrate components. Previous studies have shown that functionalized ILs can improve lignin’s separation efficiency while preserving its reactivity [9,10]. This provides a technical basis for the directed conversion of lignin into phenolic compounds and functional materials, and offers valuable insights for developing biomass-refining chains [11,12].

Owing to their low volatility and high chemical stability, ILs are considered “green solvents” for lignocellulosic biomass pretreatment [13]. Tolesa et al. investigated the degradation of alkali lignin using the ammonium-based ILs N,N-diisopropylethylamine chloride ([DIPEA][Cl]) and N,N-diisopropylethylamine benzoate ([DIPEA][Bn]) in aqueous solution at 170 °C for 60 min. They found that the ILs acted as both solvent and catalyst, cleaving key ether linkages (e.g., β-O-4) within the lignin structure [14]. Montes et al. examined the selective separation of lignin from banana stems using choline lactate ([Cho][Lac]). Under mild conditions (80 °C, 48 h), a lignin extraction yield of 15.8 wt% and a cellulose retention rate of 77.4 wt% were achieved. The delocalized charge of the lactate anion interacts with the aromatic structure of lignin, promoting the cleavage of β-O-4 linkages [15]. Rigual et al. reported that 1-ethyl-3-methylimidazolium acetate ([Emim][OAc]) efficiently cleaves lignin at 120 °C within 6 h, achieving high extraction yields. The mechanism involves hydrogen bonding between the acetate anion and phenolic hydroxyl groups, together with π–π interactions between the imidazolium cation and aromatic rings, which collectively disrupt the lignin structure and cleave β-O-4 bonds, resulting in an enzymatic digestibility of 84% [16]. Despite the widespread application of conventional ILs (e.g., imidazolium- and pyridinium-based salts) in lignin separation, they generally suffer from complex synthesis, high cost, and insufficient selectivity for lignin deconstruction. This makes it difficult to achieve both efficient dissolution and preservation of reactive activity [17].

Protic ionic liquids (PILs) have received attention due to their unique Brønsted acid-base proton transfer mechanism. These ionic liquids form spontaneously through proton exchange between an acidic component (e.g., a carboxylic acid) and a basic component (e.g., an amine). They feature straightforward synthesis (typically a one-step acid-base neutralization), inexpensive and readily available raw materials, as well as favorable solubility and environmental compatibility [18,19]. In PIL research, the mole ratio of acidic to basic components can be optimized according to pretreatment requirements; the 1:1 stoichiometric ratio merely represents the basis for forming a neat PIL and is not the only option. Nakasu et al. systematically investigated the effect of the acid-base mole ratio (ABR) over a range of 0.1 to 10 on the pretreatment of sugarcane bagasse using a monoethanolamine acetate PIL. They found that the lignin extraction yield increased with higher base (amine) content, reaching 84% (i.e., 84% of the original lignin in the raw sugarcane bagasse was removed) at an ABR of 0.1 (large excess of amine) [20]. Pin et al. systematically studied the pretreatment performance of 20 different PILs, which also involved PIL preparation and application at various stoichiometric ratios [21]. Liu et al. developed an ethanolamine acetate PIL that, when applied to isolated industrial alkali lignin, increased the phenolic hydroxyl content of the lignin by 25.3% after treatment at 70 °C for 2 h. The proposed mechanism involves enhanced ionization, which promotes nucleophilic attack of acetate ions on methoxy groups, leading to demethylation [22]. Pin et al. further investigated the effect of cation alkyl chain length on the pretreatment of sugarcane bagasse using [MEA][AA]-type PILs, observing that increasing the cation chain length raised viscosity while decreasing the degree of ionization. Among the tested systems, [MEA][OAc] (C2) exhibited the best lignin separation capability [21]. These studies demonstrate that by tuning the stoichiometry and molecular structure of PILs, it is possible to precisely control their solubility and reaction selectivity in biomass pretreatment [8,23].

Building on existing PIL research, we selected the ethanolamine pyruvate (EAP) system based on the following considerations. First, the pyruvate anion contains both a carboxyl group (proton donor) and a carbonyl group (electron acceptor). This bifunctional structure is expected to create a dynamic catalytic microenvironment that synergistically promotes β-O-4 ether bond cleavage and methoxy removal, offering advantages over monofunctional anions such as acetate [24]. Second, compared with the reported ethanolamine acetate system, EAP may achieve higher lignin separation selectivity and lower carbohydrate dissolution under mild conditions. Third, the EAP system is simple to synthesize, uses low-cost raw materials, and allows solvent recovery after pretreatment. However, to the best of our knowledge, the use of ethanolamine pyruvate as a protic ionic liquid for lignocellulosic biomass pretreatment and targeted structural modification of lignin has not yet been reported [25].

It should be noted that ethanolamine pyruvate as a compound has been documented in the literature (e.g., early studies on PILs formed from ethanolamine and carboxylic acids). Therefore, the contribution of this study is not primarily in solvent design. Using eucalyptus as the feedstock, we systematically optimized the EAP mole ratio, treatment temperature, and reaction time. The study focuses on evaluating two aspects. First, we assess the selective separation capability of EAP pretreatment for lignin, including the separation yields of lignin, cellulose, and hemicellulose. Second, we examine the structural changes in the isolated lignin, such as its phenolic hydroxyl content, molecular weight, and S/G ratio. Through these evaluations, we aim to determine whether the EAP system can achieve a balance between separation selectivity and product structural modification.

The evolution of the raw material and treated fibers in terms of microstructure, functional group changes, crystalline structure, and thermal stability was characterized using scanning electron microscopy (SEM), Fourier transform infrared spectroscopy (FTIR), X-ray diffraction (XRD), and thermogravimetric analysis (TGA). The molecular weight distribution, inter-unit linkage patterns, and functional group content (e.g., phenolic hydroxyls) of the isolated lignin were systematically analyzed using gel permeation chromatography (GPC), two-dimensional heteronuclear single-quantum coherence nuclear magnetic resonance (2D-HSQC NMR), and phosphorus-31 nuclear magnetic resonance (^31^P NMR). These characterization results are intended to verify the synergistic effect of EAP pretreatment on separation efficiency and product reactivity, thereby providing a pretreatment route that balances selective separation and structural regulation for the high-value utilization of lignin.

## 2. Materials and Methods

### 2.1. Materials

Eucalyptus was sourced from a local forest plantation in Guangxi, China. The wood was ground into powder with a particle size of 60–100 mesh and stored for subsequent use. Its chemical composition is provided in Table S1. Ethanolamine (99.5%), acetic acid (99.8%), and pyruvate (98.0%) were purchased from Aladdin (Shanghai, China). Other chemicals used included dioxane (analytical grade, 98.0%, Aladdin), acetone (analytical grade, 99.5%, Aladdin), deuterated dimethyl sulfoxide (DMSO-d_6_, 99.9%, Aladdin), chromium(III) acetylacetonate (97.0%, Sigma-Aldrich, Shanghai, China), N-hydroxysuccinimide (98.0%, Sigma-Aldrich, Shanghai, China), anhydrous pyridine (99.8%, Sigma-Aldrich, Shanghai, China), deuterated chloroform (CDCl_3_, 99.9%, Sigma-Aldrich, Shanghai, China), and 2-chloro-4,4,5,5-tetramethyl-1,3,2-dioxaphospholane (95.0%, Sigma-Aldrich, Shanghai, China).

### 2.2. Preparation of EAP and Pretreatment Separation of Eucalyptus

All experiments were performed in triplicate, and data are presented as mean ± standard deviation.

An appropriate amount of pyruvate was placed in a glass beaker. Under an ice-water bath, different masses of ethanolamine were added dropwise to achieve pyruvate-to-ethanolamine mole ratios of 1:1, 1:2, 1:3, 1:4, and 1:5. When the mole ratio is 1:1, EAP forms a neat protic ionic liquid. When ethanolamine is in excess (e.g., mole ratios of 1:2 to 1:5), the system contains excess ethanolamine and thus becomes a mixture of the protic ionic liquid and the surplus ethanolamine. The mixture was mechanically stirred at 120 rpm throughout the addition to ensure adequate heat dissipation. The reaction was allowed to proceed under continuous stirring for 12 h to complete the ion-exchange process. Excess moisture in the resulting EAP product was removed using a rotary evaporator.

Pretreatment experiments were conducted in a high-temperature oven (FDL 115, Binder, Neckarsulm, Germany). Wood powder (2 g) was placed in a 100 mL PTFE-lined vessel, and 20 mL of ethanolamine pyruvate ionic liquid solutions with varying mole ratios was added and thoroughly mixed. The reactions were performed at different temperatures (40–140 °C) and durations (10–60 min). After completion, the system was cooled to room temperature, followed by solid–liquid separation. The solid residue was washed repeatedly with distilled water until the washings became neutral, then dried in an oven at 60 °C for 24 h. Distilled water was added to the filtrate to precipitate the lignin. The precipitate was washed to neutrality and then freeze-dried. The filtrate was subsequently evaporated in a vacuum oven at 70 °C to remove water and recover the PIL. The lignin separation rate from the PIL solution was determined according to prior research [26], with the formula provided in Equation (1).

(1) $$Y_{L}=\frac{C_{S}+C_{IS}}{C_{L0}}\times 100\%$$

$Y_{L}$ denotes the lignin separation rate; $C_{L0}$ signifies the content of unpretreated lignin; $C_{S}$ represents the content of acid-soluble lignin post-pretreatment; and $C_{IS}$ indicates the content of acid-insoluble lignin post-pretreatment.

### 2.3. Calculation of PIL Pretreatment Separation Efficiency

The chemical composition of the wood before and after pretreatment was analyzed according to the standard procedures of the National Renewable Energy Laboratory (NREL). Acid-insoluble lignin separation yield was determined gravimetrically. Lignin separation yield was analyzed and calculated using a UV–visible spectrophotometer (CARY 3500, Agilent, CA, USA) (see Equations (S1)–(S3)). Monosaccharides in the hydrolysates were quantified by high-performance liquid chromatography (HPLC, Alliance E2695, Waters, MA, USA) (see Equations (S4) and (S5)). The lignin separation yield refers to the percentage of the original lignin in the raw material that is extracted into the ionic liquid phase, while the dissolution yields of cellulose and hemicellulose refer to the percentages of the original polysaccharides that are solubilized during pretreatment. These yields are calculated based on the compositional analysis before and after pretreatment, using the formulas provided in the Supplementary Information (Equations (S7)–(S9)).

Ash content was determined following the NREL standard method NREL/TP-510-42622. An oven-dried sample (2 g) was accurately weighed into a pre-weighed ceramic crucible, placed in a muffle furnace, and heated at a rate of 5–10 °C/min to 575 ± 25 °C. The temperature was maintained for 4 h. After incineration, the furnace was allowed to cool to approximately 200 °C. The crucible was then transferred to a desiccator and cooled to room temperature for about 1 h before weighing. Ash content was calculated using the following formula:

(2) $$Ash\left(\%\right)=\frac{m_{ash}-m_{sieve}}{m_{sample}}\times 100\%$$

where $m_{ash}$ is the mass of the crucible plus residue after incineration (g), $m_{sieve}$ is the mass of the empty crucible (g), and $m_{sample}$ is the mass of the oven-dried sample (g). Three independent replicate determinations were performed for each sample, and results are expressed as mean ± standard deviation.

### 2.4. Characterization of Eucalyptus After Pretreatment

The surface morphology of untreated and pretreated eucalyptus was observed using a scanning electron microscope (SEM, SU8220, Hitachi, Tokyo, Japan) at an accelerating voltage of 5 kV and a working distance of 8 mm. Before imaging, the samples were sputter-coated with gold for 30 s. Magnifications of 500× and 1000× were used.

Fourier transform infrared (FTIR) spectra were recorded on a TENSOR 27 spectrometer (Bruker, Karlsruhe, Germany) equipped with an attenuated total reflection (ATR) accessory. Spectra were collected in the range of 4000–600 cm^−1^ with a resolution of 4 cm^−1^ and 32 scans.

X-ray diffraction (XRD) patterns were obtained using a MINFLEX600 diffractometer (Rigaku, Tokyo, Japan) with Cu Kα radiation (λ = 0.15418 nm) operated at 40 kV and 40 mA. The scanning range was 2θ = 5–50° at a speed of 5°/min. The crystallinity index (CrI) was calculated according to Equation (S10) in the Supplementary Information.

Thermogravimetric analysis (TGA) was performed on a TGA55 instrument (TA Instruments, New Castle, DE, USA). Approximately 5 mg of dried sample was placed in a platinum pan and heated from room temperature to 800 °C at a rate of 10 °C/min under a N_2_ atmosphere (flow rate 50 mL/min). The thermogravimetric (TG) and derivative thermogravimetric (DTG) curves were recorded.

X-ray photoelectron spectroscopy (XPS) was conducted on an ESCALAB 250XI spectrometer (Thermo Fisher Scientific, Waltham, MA, USA) using monochromatic Al Kα radiation (hv = 1486.6 eV). The vacuum in the analysis chamber was better than 5 × 10^−10^ mbar. Survey scans were acquired with a pass energy of 100 eV, and high-resolution scans with a pass energy of 20 eV. The binding energy scale was calibrated using the C 1s peak at 284.8 eV. The surface lignin coverage (SLC) was calculated according to Equation (S11) in the Supplementary Information.

Solid-state ^13^C cross-polarization/magic-angle spinning nuclear magnetic resonance (^13^C CP/MAS NMR) spectra were recorded on an AVANCE NEO 400 WB spectrometer (Bruker, Karlsruhe, Germany) at a magnetic field of 9.4 T (^13^C resonance frequency 100.6 MHz). Samples were packed into a 4 mm ZrO_2_ rotor and spun at a magic-angle spinning rate of 8 kHz. Detailed acquisition parameters are provided in the Supplementary Information.

### 2.5. Structural Characterization of Lignin

The purity of the isolated lignin was determined by the two-step acid hydrolysis method according to NREL/TP-510-42618 (see Supplementary Information Method for detailed procedures). Elemental composition (C, H, N, S) was analyzed using a Vario EL cube elemental analyzer (Elementar, Langenselbold, Germany). The oxygen content was determined by difference. The weight-average (Mw) and number-average (Mn) molecular weights of lignin were measured by gel permeation chromatography (GPC, PL-GPC50, Waters, Milford, MA, USA) under two different mobile phase systems depending on the solubility of the lignin samples. Detailed GPC conditions and calibration standards are described in the Supplementary Information. The structural features of lignin were characterized by two-dimensional heteronuclear single-quantum coherence nuclear magnetic resonance (2D HSQC NMR) spectroscopy on an AVANCE III HD 500 MHz spectrometer (Bruker, Karlsruhe, Germany). The contents of different hydroxyl groups (aliphatic OH, phenolic OH, and carboxyl OH) were quantified by ^31^P NMR using an Avance NEO 600 spectrometer (Bruker, Karlsruhe, Germany). The detailed acquisition and processing parameters for 2D HSQC NMR and ^31^P NMR are provided in the Supplementary Information.

#### 2.5.1. Lignin Purity

The lignin purity was determined using the two-step acid hydrolysis method described by the National Renewable Energy Laboratory (NREL). In brief, 0.2 g of oven-dried lignin sample was hydrolyzed with 72% H_2_SO_4_ at 30 °C for 1 h, followed by dilution to 4% H_2_SO_4_ and autoclaving at 121 °C for 1 h. The acid-insoluble lignin (AIL) was recovered by filtration and gravimetrically quantified. The acid-soluble lignin (ASL) in the filtrate was determined by UV–Vis spectrophotometry at 205 nm. The lignin purity was calculated as the sum of AIL and ASL divided by the initial dry mass of the lignin sample. All measurements were performed in triplicate, and the results are expressed as mean ± standard deviation.

#### 2.5.2. Elemental Analysis

The contents of carbon (C), hydrogen (H), nitrogen (N), and sulfur (S) in lignin were determined using a Vario EL cube elemental analyzer (Elementar, Langenselbold, Germany). Approximately 2–3 mg of each lignin sample was combusted at 1150 °C. Helium was used as the carrier gas (200 mL/min), and oxygen was used as the combustion aid (25 mL/min). The instrument was calibrated with a sulfanilamide standard. The oxygen content was calculated by difference (O%=100%−C%−H%−N%−S%−ash%). Each sample was analyzed in triplicate, and the data are reported as mean ± standard deviation.

#### 2.5.3. Gel Permeation Chromatography (GPC)

Molecular weight determination was carried out on a PL-GPC50 system (Waters, Milford, MA, USA). For water-soluble lignin (e.g., lignin extracted by EAP), an aqueous mobile phase (0.1 M NaOH containing 0.02% NaN_3_) was used with a PolarGel-M column (300 × 7.5 mm, Agilent, Santa Clara, CA, USA) at 40 °C and a flow rate of 0.5 mL/min. For organic-soluble lignin (e.g., milled wood lignin), a THF-based system was employed with a Phenogel column (300 × 7.8 mm, 5 μm, Phenomenex, Torrance, CA, USA) at 35 °C and a flow rate of 1.0 mL/min. Calibration was performed using narrow-distribution poly(sodium styrenesulfonate) standards (aqueous system) or polystyrene standards (THF system). The detailed calibration ranges and additional parameters are provided in the Supplementary Information. Each sample was measured in triplicate, and the results are expressed as mean ± standard deviation.

#### 2.5.4. Two-Dimensional NMR (2D HSQC NMR)

Then, 2D HSQC NMR spectra were acquired on an AVANCE III HD 500 MHz spectrometer (Bruker, Karlsruhe, Germany) at 25 °C using DMSO-d_6_ as the solvent. Approximately 80 mg of lignin was dissolved in 700 μL of DMSO-d_6_. The acquisition parameters (e.g., number of scans, relaxation delay, spectral widths) are detailed in the Supplementary Information. Semi-quantification of lignin structural units (β-O-4, β-β, β-5, S/G ratio) was performed by integrating the corresponding signals in the HSQC spectra according to the equations provided in the Supplementary Information. All samples were analyzed in triplicate, and the data are expressed as mean ± standard deviation.

#### 2.5.5. ^31^P NMR for Hydroxyl Group Quantification

^31^P NMR spectra were recorded on an Avance NEO 600 spectrometer (Bruker, Karlsruhe, Germany) after derivatization of lignin with 2-chloro-4,4,5,5-tetramethyl-1,3,2-dioxaphospholane (Cl-TMDP). A stock solution containing chromium(III) acetylacetonate (relaxation reagent, 50 mmol/L) and N-hydroxysuccinimide (internal standard, 50 mmol/L) in anhydrous pyridine was prepared. Typically, 20 mg of dried lignin was mixed with 400 μL of CDCl_3_ and 200 μL of the stock solution, followed by the addition of 80 μL of Cl-TMDP. The reaction mixture was then transferred to a 5 mm NMR tube. The acquisition parameters (e.g., pulse sequence, number of scans, relaxation delay) are provided in the Supplementary Information. The contents of aliphatic OH, phenolic OH, and carboxyl OH were calculated by comparing the integrated signal areas of the derivatized hydroxyl groups with that of the internal standard. Each sample was analyzed in triplicate, and the results are expressed as mean ± standard deviation.

#### 2.5.6. Antioxidant Activity

The antioxidant activity of the isolated lignin was evaluated using the 2,2-diphenyl-1-picrylhydrazyl (DPPH) radical scavenging assay. Lignin samples were dissolved in dioxane/water (9/1, *v*/*v*) at concentrations ranging from 0.05 to 0.5 mg/mL. An aliquot (0.1 mL) of the lignin solution was mixed with 3.9 mL of a 25 mg/mL DPPH–ethanol solution. The mixture was incubated in the dark at room temperature for 30 min. The absorbance was measured at 517 nm using a UV–Vis spectrophotometer, with absolute ethanol as the blank. The DPPH radical scavenging activity was calculated as

$$DPPH\;radical(\%)=\frac{A_{0}-A_{1}}{A_{0}}$$

where $A_{0}$ and $A_{1}$ are the absorbances of the blank and the sample, respectively. The IC_50_ value (the concentration required to scavenge 50% of DPPH radicals) was determined from the dose–response curve.

## 3. Results and Discussion

### 3.1. Efficient Separation of Eucalyptus Lignin by PIL Pretreatment

**Figure 1:**
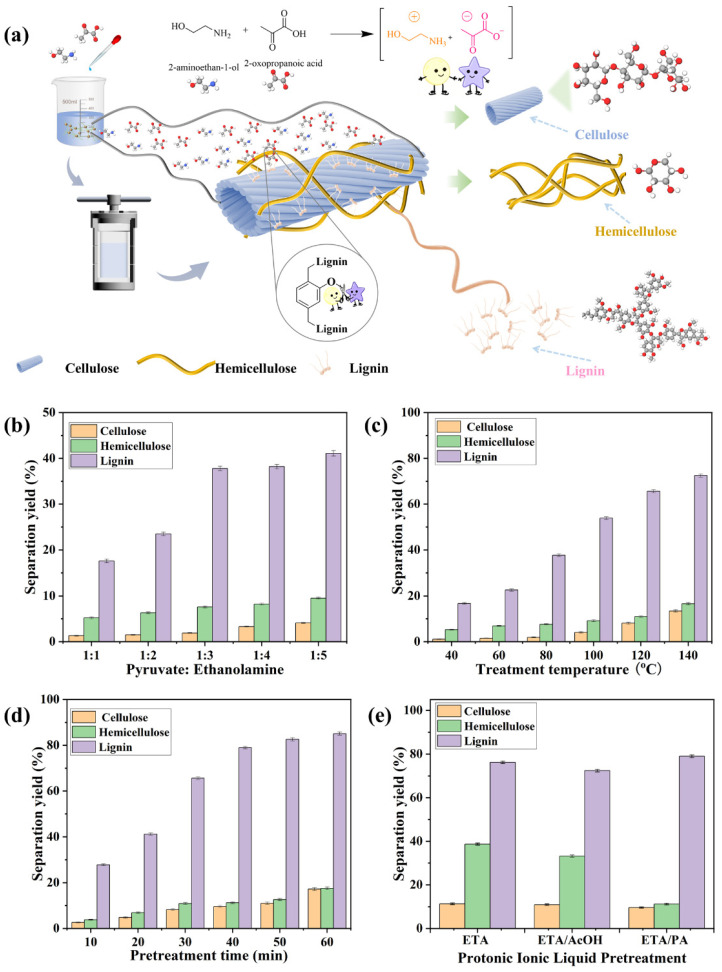
Effects of ethanolamine pyruvate (EAP) pretreatment on the separation of eucalyptus components. (**a**) Separation schematic diagram. (**b**) Effect of mole ratio (1:1 to 1:5) at 80 °C for 30 min. (**c**) Effect of temperature (40–140 °C) at a mole ratio of 1:3 for 30 min. (**d**) Effect of time (10–60 min) at a mole ratio of 1:3 and 120 °C. (**e**) Comparison of EAP with ethanolamine (ETA) and ethanolamine acetate (ETA/AcOH) under their respective optimal conditions. Separation yield is defined as the mass percentage of a component (lignin, cellulose, or hemicellulose) extracted from the raw material relative to its initial mass in the raw material.

The proton transfer between the carboxyl and amino groups in EAP enables the simultaneous separation and chemical activation of lignin (Figure 1a). The EAP mole ratio is a key factor determining the acid–base properties and separation selectivity during process optimization, as it regulates the pH and alkalinity of the system, thereby altering solvent solubility and affecting lignin dissolution. Accordingly, the effect of the EAP mole ratio on selective lignin separation was investigated at 80 °C for 30 min using pyruvate-to-ethanolamine mole ratios of 1:1, 1:2, 1:3, 1:4, and 1:5. At a 1:1 mole ratio, EAP forms a neat protic ionic liquid. When ethanolamine is in excess (e.g., mole ratios of 1:2 to 1:5), the system contains excess ethanolamine and thus becomes a mixture of the protic ionic liquid and the surplus ethanolamine. The excess ethanolamine plays an important synergistic role during pretreatment: its free amine groups form hydrogen-bonding networks with the phenolic hydroxyl groups of lignin, enhancing lignin dissolution; meanwhile, the alkaline environment it provides helps stabilize reactive intermediates and suppress lignin recondensation side reactions.

The separation yields of cellulose, hemicellulose, and lignin as a function of the EAP mole ratio are shown in Figure 1b. All experiments were performed in triplicate, and results are expressed as mean ± standard deviation. When the ethanolamine-to-pyruvate mole ratio increased from 1:1 to 1:3, the lignin separation yield increased from 17.6 ± 0.4% to 37.8 ± 0.5%. When the mole ratio was further increased from 1:3 to 1:5, the lignin yield rose from 37.8 ± 0.5% to 41.1 ± 0.6%, an increase of approximately 3.3 percentage points. The improvement in lignin separation is primarily attributed to two factors. First, the amine groups of the ethanolamine cation form an extensive hydrogen-bonding network with the phenolic hydroxyl groups of lignin, effectively disrupting the native linkages between lignin and carbohydrates [27]. Second, the increased alkalinity of the system further promotes lignin dissolution [28]. Simultaneously, the pyruvate anion plays a key role, as its carbonyl and carboxyl groups contribute to the initial cleavage of β-O-4 ether linkages via dipole–charge interactions [29]. When the mole ratio exceeded 1:3, the lignin separation yield increased only marginally (by about 3.3 percentage points from 1:3 to 1:5), suggesting that the hydrogen-bond-promoting effect of ethanolamine approached saturation and that the accessible binding sites in lignin were nearly exhausted [30]. Meanwhile, the cellulose separation yield increased from 1.9 ± 0.1% to 4.1 ± 0.1%, a relative increase of approximately 116%, indicating that an excessively alkaline environment may disrupt the crystalline structure of cellulose. From a mechanistic perspective, excess ethanolamine could not only disrupt the internal hydrogen-bonding network of cellulose but also, due to its strong alkalinity, promote the hydrolysis of glycosidic bonds, resulting in irreversible degradation of cellulose [31]. The results demonstrate that the synergistic interaction between the EAP cation and anion provides an advantage under various treatment conditions [32]. Consequently, an EAP mole ratio of 1:3 was identified as optimal, as it achieves a balance between efficient lignin separation and maximal preservation of cellulose and hemicellulose structural integrity.

The effect of reaction temperature on component separation performance was investigated at a fixed mole ratio of 1:3 and a reaction time of 30 min. All experiments were performed in triplicate, and results are expressed as mean ± standard deviation. As shown in Figure 1c, when the temperature increased from 40 °C to 120 °C, the lignin separation yield increased from 16.7 ± 0.4% to 65.6 ± 0.7%. This enhancement is attributed to the multifaceted influence of temperature on the physicochemical properties and catalytic activity of the ionic liquid. Elevated temperature not only reduced the system viscosity, thereby improving the penetration of the ionic liquid into the lignocellulosic matrix, but also activated the catalytic function of the pyruvate anion [33]. Under thermal activation, its unique carbonyl and carboxyl groups more effectively cleave β-O-4 ether linkages in lignin. Furthermore, a portion of pyruvate converts to acetic acid upon heating, and the resulting acid–base equilibrium further optimizes the conditions for lignin dissolution. However, when the temperature reached 140 °C, the system selectivity decreased markedly. The lignin separation yield increased from 65.6 ± 0.7% at 120 °C to 72.5 ± 0.7% at 140 °C, an increase of approximately 6.9 percentage points; meanwhile, the dissolution yields of cellulose and hemicellulose rose to 13.4 ± 0.5% and 16.6 ± 0.5%, respectively. This loss of selectivity is attributed to excessive pyrolysis, which catalyzes the extensive conversion of pyruvate to acetic acid. The consequent increase in system acidity accelerates the hydrolysis of glycosidic bonds in carbohydrates. Under the combined effects of high temperature and elevated acidity, cellulose undergoes structural damage, while hemicellulose—the least thermally stable component—experiences more severe degradation [34]. Considering both depolymerization efficiency and the preservation of component structures, 120 °C was considered the optimal reaction temperature.

Figure 1d presents the temporal variation in eucalyptus component separation at a fixed mole ratio of 1:3 and 120 °C. All experiments were performed in triplicate, and results are expressed as mean ± standard deviation. The lignin separation yield increased rapidly from 27.8 ± 0.4% at 10 min to 79.0 ± 0.6% at 40 min, an increase of approximately 51.2 percentage points. Beyond 40 min, the rate of increase slowed markedly, with the lignin yield reaching 85.1 ± 0.7% at 60 min, an increase of about 6.1 percentage points relative to the 40 min value. This trend is attributed to the EAP achieving sufficient penetration and cleaving the ether linkages within lignin within the initial 40 min, leading to a high dissolution rate. After this period, the available cleavable bond sites became largely depleted, causing the lignin dissolution process to approach saturation. Concurrently, the separation yields of cellulose and hemicellulose increased from 2.6 ± 0.2% and 3.8 ± 0.2% at 10 min to 9.6 ± 0.4% and 11.2 ± 0.4% at 40 min, respectively. Between 40 and 60 min, the cellulose yield rose from 9.6 ± 0.4% to 17.2 ± 0.6% (an increase of about 7.6 percentage points), and the hemicellulose yield rose from 11.2 ± 0.4% to 17.5 ± 0.6% (an increase of about 6.3 percentage points). This secondary rise is due to the continuous conversion of pyruvate to acetic acid, which elevates system acidity and intensifies glycosidic bond hydrolysis [35]. Therefore, a reaction time of 40 min is sufficient to achieve efficient lignin separation while minimizing carbohydrate loss, establishing it as the optimal duration for EAP pretreatment.

In summary, the optimal process conditions for EAP pretreatment were determined as a pyruvate-to-ethanolamine mole ratio of 1:3, a temperature of 120 °C, and a duration of 40 min. Under these conditions, the lignin separation yield was 79.0 ± 0.6%, while the separation yields of cellulose and hemicellulose were 9.6 ± 0.3% and 11.2 ± 0.4%, respectively. Furthermore, the separation performance of EAP was compared with those of ethanolamine acetate (ETA/AcOH) and pure ethanolamine (ETA). All experiments were performed in triplicate, and results are expressed as mean ± standard deviation. Pure ethanolamine pretreatment achieved a lignin separation yield of 76.2 ± 0.6%, but resulted in a high hemicellulose separation yield of 38.7 ± 0.5% and a cellulose separation yield of 11.3 ± 0.4%. Ethanolamine acetate pretreatment gave a lignin yield of 72.4 ± 0.6%, a hemicellulose yield of 33.2 ± 0.5%, and a cellulose yield of 10.9 ± 0.4%. Among the three systems, EAP exhibited the highest lignin separation yield and the lowest dissolution of cellulose and hemicellulose. This outcome may be attributed to the bifunctional structure of the pyruvate anion, which contains both carbonyl and carboxyl groups. Through enhanced dipole–charge interactions and catalytic activity, the pyruvate anion may facilitate the cleavage of β-O-4 ether linkages in lignin while suppressing the degradation of carbohydrates [36]. In contrast, the acetate anion relies solely on a single carboxyl group, resulting in lower separation selectivity. Consequently, the ethanolamine pyruvate (EAP) system, leveraging the synergy between ethanolamine and pyruvate, achieves selective lignin separation under relatively mild conditions.

**Figure 2:**
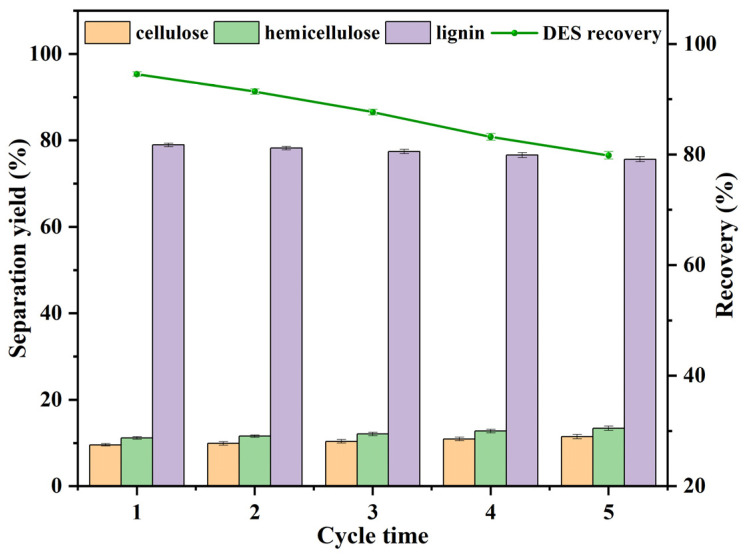
Recycling performance of the EAP solvent: separation yields of cellulose, hemicellulose, and lignin at different cycle numbers (1–5), and solvent recovery rate.

The recovery rate of the EAP system is a key parameter for evaluating process economics. Thus, its recyclability was assessed (Figure 2). The recovery rate ranged from 79.8 ± 0.7% to 94.5 ± 0.4%. After five reuse cycles, the lignin separation yield remained at 75.6%. Although the recovery rate gradually decreased with increasing cycle number—likely due to physical loss and minor degradation of active components—the lignin separation yield declined by only 3.4 percentage points. This indicates that the EAP system possesses acceptable reusability and renewability potential.

The ash content in the solid residue after EAP pretreatment was 2.3 ± 0.1%, lower than that of the raw material (3.4 ± 0.1%) (Table 1). This indicates that the pretreatment process removed a portion of the inorganic components from the feedstock. The ash removal rate after EAP pretreatment was approximately 32.4%, which is attributed to the dissolution of soluble inorganic salts by the acidic component (pyruvate) during pretreatment and to the decrease in solid recovery, where some ash was removed along with the solubilized components. A certain amount of ash remains in the solid after pretreatment. This residual ash likely originates from structurally bound inorganic species in the raw material, such as calcium oxalate and silica. The lower ash content is beneficial for improving the purity of the isolated lignin and reducing potential interference from inorganic impurities during subsequent thermochemical conversion or functional material applications [37]. In contrast, excessive ash residues may introduce additional impurities, thereby affecting the lignin product quality and its performance in high-value applications.

### 3.2. High Preservation of Eucalyptus Fibers by EAP Pretreatment

**Figure 3:**
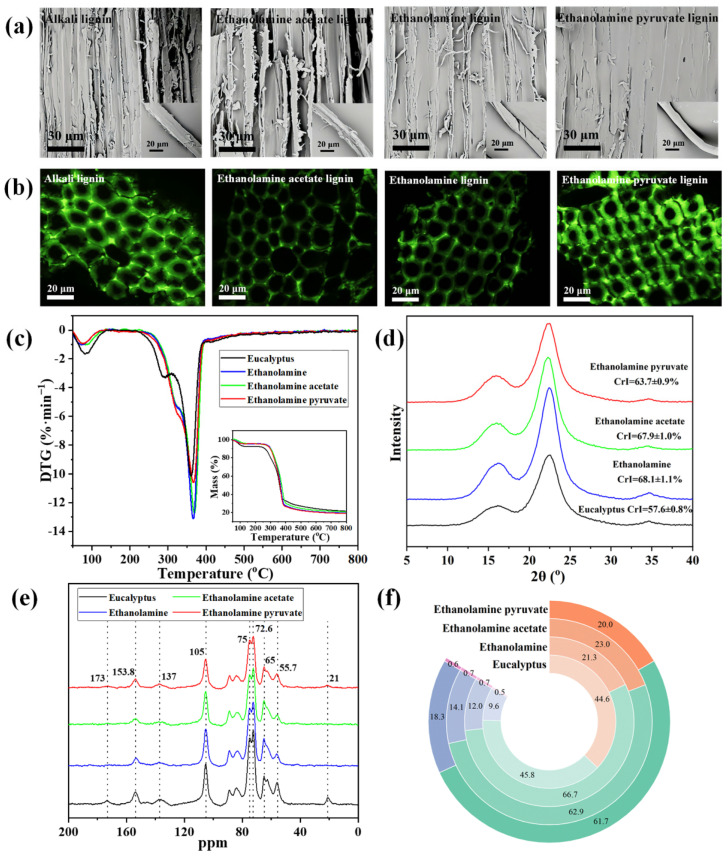
Changes in the physicochemical structure of eucalyptus fiber before and after different PIL pretreatments. (**a**) SEM: EAP-treated fibers remained intact and smooth, whereas ETA and ETA/AcOH caused severe structural damage (fibrillation or partial fracture). (**b**) CLSM: EAP preserved hemicellulose fluorescence to a greater extent than the other two systems. (**c**) TGA: the EAP-treated residue showed the lowest maximum weight loss rate (10.5 ± 0.3%·min^−1^), indicating better cellulose integrity. (**d**) XRD: the crystallinity index (CrI) followed the order raw (57.6 ± 0.8%) < EAP (63.7 ± 0.9%) < ETA/AcOH (67.9 ± 1.0%) < ETA (68.1 ± 1.1%). (**e**) ^13^C-CP/MAS-NMR: EAP removed lignin while causing less damage to hemicellulose side chains (signals at 21 and 173 ppm). (**f**) XPS: EAP resulted in the largest decrease in C1 content (24.6%) and the smallest increase in O/C ratio (0.13), confirming its higher selectivity.

Figure 3a shows that the raw sample exhibits a dense three-dimensional network structure formed by the cross-linking of cellulose, hemicellulose, and lignin, which is characteristic of natural woody fiber composites. Although traditional ethanolamine pretreatment removes lignin, its aggressive nature causes severe disintegration of the cell wall, resulting in pronounced fibrillation and compromising the material’s potential for subsequent utilization [38]. Ethanolamine acetate pretreatment is relatively mild and produces smoother surfaces, but it still induces considerable polysaccharide leaching and localized fiber breakage, reflecting insufficient selectivity [39]. EAP pretreatment yields the best results: the fibers exhibit only slight shrinkage without cracks or breaks, maintaining overall integrity and distinct fiber bundles. This outcome confirms that EAP removes lignin while preserving the fibrous skeleton, providing a robust foundation for the high-value utilization of biomass.

As shown in Figure 3b, treatments with traditional ethanolamine and acetate systems caused a significant reduction in hemicellulose separation yield and a marked decrease in fluorescence intensity, indicative of their non-selective dissolution of polysaccharides. Conversely, the EAP system demonstrated good selectivity. The corresponding samples showed minimal disturbance to hemicellulose, with some localized regions even exhibiting enhanced fluorescence—a phenomenon attributed to the increased relative content of hemicellulose in the residual solids after selective lignin removal. Thus, although traditional ethanolamine and ethanolamine acetate pretreatments separate lignin, they concurrently cause substantial dissolution of cellulose and hemicellulose, severely damaging the residual fiber structure. In contrast, EAP pretreatment, by virtue of its high selectivity for lignin, removes lignin while preserving the structural integrity of cellulose and hemicellulose, thereby enhancing the physicochemical stability and subsequent utilization potential of the residual solids.

Figure S1 shows that the characteristic absorption peaks at 1600 cm^−1^ and 1246 cm^−1^ correspond to the benzene ring skeleton vibration and the ether bond stretching vibration of lignin, respectively. Their intensities decreased after pretreatment, indicating lignin dissolution. Additionally, the absorption peak at 1740 cm^−1^ is attributed to the C=O stretching vibration of acetyl groups in hemicellulose. Following conventional ethanolamine and ethanolamine acetate treatments, the intensity of this peak decreased markedly, consistent with hemicellulose degradation and dissolution. Notably, this peak was better preserved in the EAP-pretreated samples, demonstrating minimal disruption to the hemicellulose structure [40].

The absorption peaks at 2900 cm^−1^ and 896 cm^−1^ are primarily ascribed to the C–H and glycosidic bond stretching vibrations in cellulose, respectively. Their enhanced intensity after pretreatment results from the removal of lignin and hemicellulose, which increases the relative cellulose separation yield. The broad peak at 3425 cm^−1^ corresponds to the O–H stretching vibration of cellulose hydroxyl groups. Following ethanolamine treatment, the intensity of this peak decreased, indicating that some hydroxyl groups underwent amination. In contrast, after EAP pretreatment, the peak intensity increased, reflecting both a higher relative cellulose separation yield and the absence of significant chemical modification to these functional groups.

Figure 3c presents the thermogravimetric (TG) curves of the raw material and the treated residual solids. All TGA measurements were performed in triplicate (*n* = 3), and results are expressed as mean ± standard deviation. The mass loss below 100 °C is primarily due to moisture evaporation. The characteristic pyrolysis temperature ranges for cellulose, hemicellulose, and lignin are 265–391 °C, 180–382 °C, and 172–525 °C, respectively. Owing to the partial dissolution of lignin and hemicellulose, the initial pyrolysis temperature of all treated samples increased. The maximum weight loss was enhanced: ethanolamine treatment gave 75.8 ± 0.8%, ethanolamine acetate treatment gave 74.3 ± 0.7%, and EAP treatment gave 76.4 ± 0.7%, representing increases of approximately 4.2, 2.7, and 4.8 percentage points, respectively, compared with the raw material (71.6 ± 0.7%). This shift is attributed to the increased relative cellulose separation yield, leading to a more concentrated pyrolysis process.

The derivative thermogravimetry (DTG) peak corresponding to cellulose decomposition intensified with higher cellulose separation yield. The maximum weight loss rates were: raw material (10.0 ± 0.3%·min^−1^), ethanolamine (13.1 ± 0.4%·min^−1^), ethanolamine acetate (12.6 ± 0.4%·min^−1^), and EAP (10.5 ± 0.3%·min^−1^). The low-temperature shoulder in the DTG curve represents hemicellulose pyrolysis. This shoulder weakened and shifted to higher temperatures after pretreatment, with more pronounced weakening observed for the ethanolamine and ethanolamine acetate treatments, indicating greater hemicellulose loss. These results demonstrate that EAP pretreatment removes lignin while better preserving hemicellulose, resulting in a higher cellulose separation yield in the residual solid compared with the other two systems.

The dissolution of lignin and hemicellulose affects the crystallinity index (CrI) of the residual solid. Pretreatment disrupts the cell wall structure while also altering the crystalline state of cellulose [41]. All XRD measurements were performed in triplicate (*n* = 3), and results are expressed as mean ± standard deviation. Figure 3d presents the XRD analysis results: the CrI of the raw material was 57.6 ± 0.8%, which changed to 63.7 ± 0.9% after EAP treatment, 67.9 ± 1.0% after ethanolamine acetate treatment, and 68.1 ± 1.1% after ethanolamine treatment. These results indicate that the CrI value after EAP pretreatment was lower than those after ethanolamine and ethanolamine acetate treatments, which may be related to the retention of hemicellulose by EAP pretreatment.

In Figure 3e, the peaks at 153.8 ppm and 137 ppm correspond to the C1/C2 aromatic carbons of lignin, while the signal at 55.7 ppm is assigned to the methoxy (–OCH_3_) group. The decrease in their intensities after treatment confirms the dissolution of lignin. The concurrent slight weakening of the peak at 75 ppm provides supporting evidence for lignin removal. Conversely, the characteristic cellulose signals (e.g., 65 ppm, 72.6 ppm) showed increased intensity, reflecting its higher relative content. The characteristic hemicellulose signals—specifically the acetyl methyl carbon at 21 ppm and the carboxyl carbon at 173 ppm—exhibited reduced intensity, indicating partial dissolution. While all three pretreatment methods achieved lignin separation, ethanolamine and ethanolamine acetate treatments caused greater hemicellulose loss, particularly involving removal of acetyl groups [42]. Among them, the reduction in hemicellulose signals after EAP treatment was less pronounced, indicating that its side-chain structure was preserved to a greater extent. In summary, EAP showed improved preservation of the hemicellulose structure compared with the other two systems during lignin removal [43].

The removal of lignin led to a decrease in the C1 content of the residual solid. All XPS measurements were performed in triplicate (*n* = 3), and results are expressed as mean ± standard deviation. As shown in Figure 3f, EAP pretreatment reduced C1 by 24.6%, ethanolamine treatment by 23.2%, and ethanolamine acetate treatment by 21.5%. With lignin dissolution, the relative contents of cellulose and hemicellulose increased, resulting in higher proportions of both C2 and C3. After EAP pretreatment, C2 increased from 45.8% to 61.7%, and C3 rose from 9.6% to 18.3%. In contrast, ethanolamine and ethanolamine acetate treatments decreased C3 by 6.3% and 4.2%, respectively, while increasing C2 by 5.0% and 1.2%, indicating greater hemicellulose loss with these conventional treatments. Cellulose is rich in hydroxyl groups and has high oxygen content; lignin and hemicellulose possess higher carbon content [44]. Following lignin removal, the oxygen-to-carbon (O/C) ratio increased by 0.13 for EAP treatment, 0.20 for ethanolamine treatment, and 0.16 for ethanolamine acetate treatment. These results suggest poorer separation selectivity for the latter two methods, accompanied by degradation of non-target components, which is consistent with the changes in C2 peak intensity. In conclusion, EAP retained more cellulose and hemicellulose during lignin removal than the other two systems.

### 3.3. Structural Comparison of Lignin Before and After EAP Pretreatment

**Figure 4:**
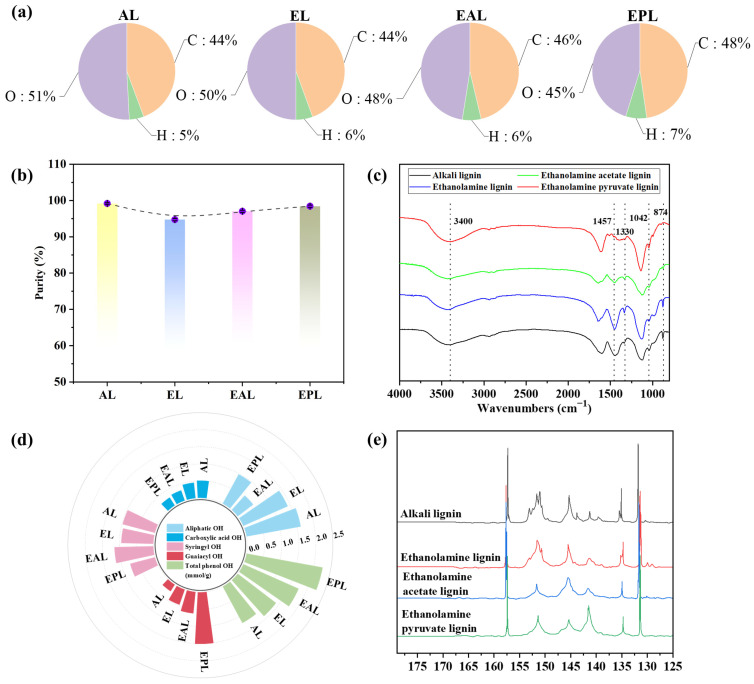
Variations in lignin separation yield and chemical structure after different PIL pretreatments. (**a**) Elemental composition (C, H, O): among the isolated lignins, EAP lignin (EPL) had the highest carbon content (48%) and the lowest oxygen content (45%). (**b**) Purity: EPL (98.4 ± 0.3%) was higher than EL (94.7 ± 0.3%) and EAL (97.0 ± 0.3%). (**c**) FTIR: EPL showed the strongest hydroxyl peak (3400 cm^−1^) and the weakest methoxy peak (1457 cm^−1^), indicating demethylation. (**d**) Hydroxyl contents determined by ^31^P NMR: total phenolic OH content (mean ± SD, mmol/g) was 1.21 ± 0.05 for AL, 1.41 ± 0.06 for EL, 1.67 ± 0.07 for EAL, and 2.26 ± 0.08 for EPL. (**e**) ^31^P NMR: EPL had the highest G-type phenolic OH content (1.51 ± 0.06 mmol/g) and the lowest S-type content (0.75 ± 0.04 mmol/g).

The composition and purity of the isolated lignin samples are presented in Figure 4a,b. All lignin purity determinations were performed in triplicate, and results are expressed as mean ± standard deviation. The purities of the four lignin samples were as follows: alkali lignin (AL) (99.2 ± 0.2%), ethanolamine lignin (EL) (94.7 ± 0.3%), ethanolamine acetate lignin (EAL) (97.0 ± 0.3%), and EAP lignin (EPL) (98.4 ± 0.3%). The dissolution of some carbohydrates during pretreatment, followed by their entrapment within the lignin during the antisolvent precipitation step, slightly reduced the final purity [45]. The purity of EAP lignin was slightly higher than that of EL and EAL, which is consistent with the lower hemicellulose dissolution observed for EAP pretreatment. Elemental analysis showed that EL and EAL had higher oxygen contents and lower carbon contents. In contrast, EAP lignin exhibited lower oxygen content and higher carbon content than the other two lignins, indicating fewer carbohydrate residues and thus higher chemical purity.

Figure 4c reveals that EAP lignin exhibited the weakest intensity at the carbohydrate characteristic peak (874 cm^−1^), indicating the lowest sugar impurity content and the highest purity. This finding is consistent with the purity analysis results discussed above. The relatively strong intensity of the hydroxyl stretching vibration peak at 3400 cm^−1^ suggests that this lignin possesses a higher hydroxyl content, which is attributed to demethylation reactions occurring during the EAP pretreatment [46]. The reduced intensity of the characteristic methoxy peak at 1457 cm^−1^ confirms demethylation, as evidenced by the lower methoxy content in EAP and EAL. Additionally, the intensity of the characteristic syringyl (S-unit) peak at 1330 cm^−1^ decreased, whereas that of the characteristic guaiacyl (G-unit) peak at 1042 cm^−1^ increased [47]. This indicates that EAP pretreatment promotes the structural transformation from S-units to G-units, a change closely linked to its demethylation capability. These findings validate the efficacy of this pretreatment method in modifying the chemical structure of lignin.

The ^31^P NMR analysis results for the lignin samples are presented in Figure 4d,e. All measurements were performed in triplicate (*n* = 3), and results are expressed as mean ± standard deviation. Lignin obtained via ionic liquid treatment showed lower aliphatic hydroxyl and carboxyl contents. Regarding phenolic hydroxyl composition, EAP lignin exhibited the highest content of G-type phenolic hydroxyl groups (1.51 ± 0.06 mmol/g) and the lowest content of S-type phenolic hydroxyl groups (0.75 ± 0.04 mmol/g). The total phenolic hydroxyl content followed the order AL (1.21 ± 0.05 mmol/g) < EL (1.41 ± 0.06 mmol/g) < EAL (1.67 ± 0.07 mmol/g) < EAP lignin (2.26 ± 0.08 mmol/g). The total phenolic hydroxyl content showed a negative correlation with the number-average molecular weight. These data indicate that after ionic liquid treatment, the phenolic hydroxyl content of lignin increased while the molecular weight decreased, which is consistent with the reported characteristics of β-O-4 ether bond cleavage and demethylation [48]. Among the samples, EAP lignin had the highest total phenolic hydroxyl content (2.26 ± 0.08 mmol/g), and its G-type phenolic hydroxyl content was higher than its S-type content, in agreement with the FTIR observations of decreased S-unit peak intensity and increased G-unit peak intensity.

### 3.4. Depolymerization and Targeted Structural Transformation of Lignin by EAP

**Figure 5:**
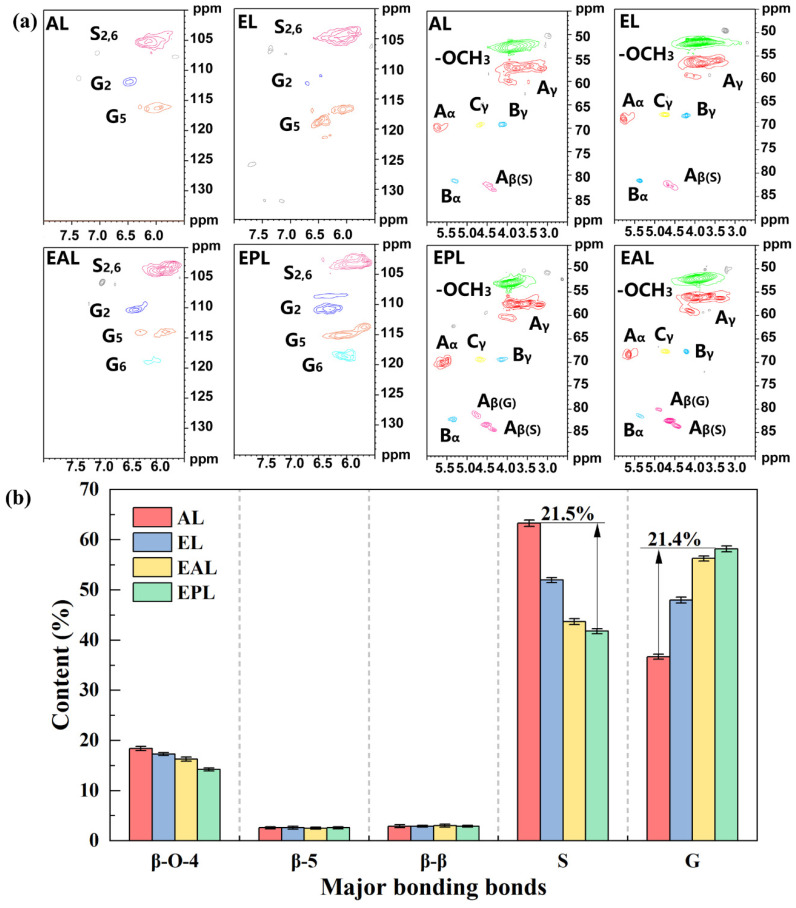
Main structural unit signals (**a**) and relative content changes (**b**) of lignin isolated by different PIL pretreatments, as determined by 2D-HSQC NMR.

To investigate the effects of different pretreatment methods on lignin structure, the structural units and inter-unit linkages of the isolated lignin were analyzed by two-dimensional heteronuclear single-quantum coherence nuclear magnetic resonance (2D-HSQC NMR) spectroscopy (Figure 5). All measurements were performed in triplicate (*n* = 3), and results are expressed as mean ± standard deviation. The results show that the isolated lignin consists primarily of syringyl (S) and guaiacyl (G) units, which is consistent with reports from the literature [49]. Compared with AL, the lignin after EAP treatment exhibited a weakened β-O-4 ether linkage signal in the HSQC spectra. The relative content of β-O-4 ether linkages decreased from 18.4 ± 0.4% in the raw lignin to 14.2 ± 0.3%, corresponding to a reduction of approximately 23%. This observation indicates that EAP treatment promotes the cleavage of β-O-4 linkages in the lignin backbone, thereby degrading lignin into lower-molecular-weight fragments.

Based on experimental observations, a plausible mechanism for the roles of each component in the EAP system can be proposed. The ethanolamine component may form hydrogen bonds with the phenolic hydroxyl groups or ether oxygen atoms of lignin, which helps disrupt the three-dimensional cross-linked structure of the lignin macromolecule. In addition, the mildly alkaline environment provided by ethanolamine may stabilize reactive intermediates generated during lignin depolymerization, thereby partially suppressing recondensation side reactions. The pyruvate component, containing both carboxyl and carbonyl groups, may promote the cleavage of the Cβ–O bond in β-O-4 ether linkages through proton catalysis. It has been reported that carboxylate anions with similar structures can participate in ether bond cleavage via proton transfer and nucleophilic action [50].

As shown in Figure 5, with the depolymerization of lignin, the relative content of S-units decreased from 63.3 ± 0.6% to 41.8 ± 0.5%, a reduction of approximately 21.5 percentage points. The relative content of G-units increased correspondingly, and the S/G ratio declined from 1.72 ± 0.05 to 0.71 ± 0.03. Meanwhile, quantitative ^31^P NMR analysis showed that the total phenolic hydroxyl content of the isolated lignin was 2.26 ± 0.08 mmol/g, and the phenolic hydroxyl characteristic peak in the FT-IR spectrum was enhanced. These observations are consistent with reported features of lignin demethylation [51]. It is thus postulated that demethylation (i.e., the removal of methoxy groups) occurred during EAP treatment, leading to the conversion of some S-units to G-units and the release of new phenolic hydroxyl groups. The mildly alkaline environment provided by ethanolamine may help stabilize the phenolic hydroxyl intermediates generated during the reaction.

**Figure 6:**
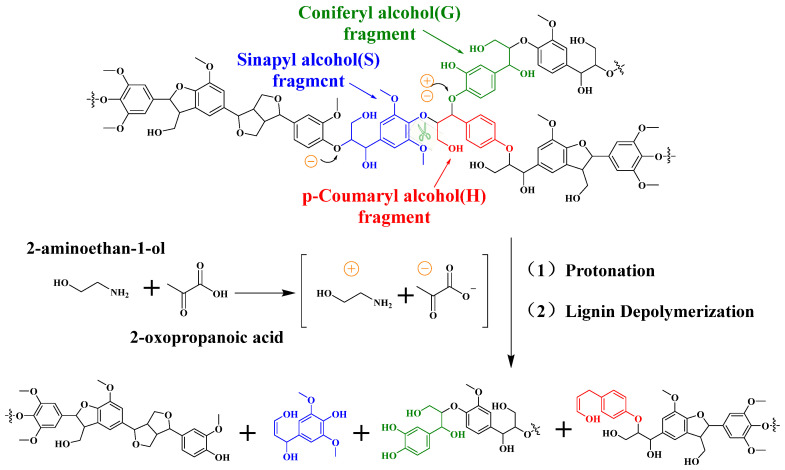
Proposed mechanism for the depolymerization and catalytic activation of lignin in eucalyptus by EAP.

Based on the above observations, EAP treatment enables simultaneous lignin depolymerization and structural modification (reduction of β-O-4 linkages, increase in phenolic hydroxyl groups, and decrease in S/G ratio) under mild conditions [52]. Ethanolamine and pyruvate likely play complementary roles in the depolymerization and functional group transformation processes: the former facilitates lignin network disruption and intermediate stabilization via hydrogen bonding and an alkaline environment, while the latter promotes ether bond cleavage and methoxy removal through proton catalysis (Figure 6). The proposed mechanism is a reasonable interpretation based on characterization results such as NMR and GPC. The decrease in β-O-4 content could be due to both ether bond cleavage and/or relative enrichment of other structural units. The increase in phenolic hydroxyl groups might also partially result from the dissociation of lignin–carbohydrate complexes [53]. Confirmation of the specific roles of each component in the EAP system awaits further studies using model compounds and intermediate capture analysis.

### 3.5. Comparison of EAP with Different Ethanolamine-Based PIL Systems

To contextualize the performance of EAP within the literature on ethanolamine-based protic ionic liquids, Table 2 summarizes the pretreatment conditions and key results from representative studies alongside the present work. Nakasu et al. pretreated sugarcane bagasse with monoethanolamine acetate ionic liquid ([MEA][OAc]) at 150 °C for 2 h, achieving a lignin extraction yield of 84% [20]. Huang et al. pretreated poplar wood using an integrated acetic acid–ethanolamine acetate (HAc-[EOA][OAc]) system. Under optimal conditions (first HAc catalysis at 170 °C for 0.5 h, followed by [EOA][OAc] pretreatment at 140 °C for 3 h), they achieved a lignin extraction yield of approximately 46% and removed about 88% of the hemicellulose [29]. Zhao et al. reported that ethanolamine acetate ionic liquid increased the phenolic hydroxyl content of technical lignin to 2.20 mmol/g or by 215.69%, respectively [24]. Li et al. treated technical lignin with a dicarboxylic acid-based ethanolamine ionic liquid ([EOA][GA]), increasing the polyphenol content by a factor of 1.58, while also observing β-O-4 bond cleavage and a decrease in the S/G ratio [54]. Rigual et al. treated eucalyptus wood with a one-pot protic ionic liquid [2-HEA][OAc] pretreatment and saccharification system (150 °C, 6 h, 10 wt% biomass loading), achieving approximately 75% glucan digestibility and 34.6% lignin removal, while also observing the growth of Rhodosporidium toruloides at IL concentrations ≤ 5 *w*/*w*% and a decrease in the S/G ratio of the residual lignin [55].

Compared with the above reports, EAP pretreatment exhibits the following characteristics (Table 3). First, at 120 °C for 40 min, the lignin separation yield of EAP was 79.0 ± 0.5%, which is close to the 84% reported by Nakasu et al. Second, the dissolution yields of cellulose and hemicellulose after EAP pretreatment were 9.6 ± 0.3% and 11.2 ± 0.4%, respectively, which are lower than the hemicellulose removal rate (approximately 88%) reported by Huang et al., indicating that EAP better preserves carbohydrates. Third, the phenolic hydroxyl content of EAP lignin was 2.26 ± 0.08 mmol/g, and the S/G ratio decreased from 1.72 ± 0.05 to 0.71 ± 0.03, which is comparable to the best results reported in the literature. It should be noted that these comparisons are qualitative due to differences in feedstock, pretreatment conditions, and analytical methods among studies. Overall, the EAP system achieves both selective lignin separation and structural modification under mild conditions.

### 3.6. Antioxidant Activity Analysis of Lignin

**Figure 7:**
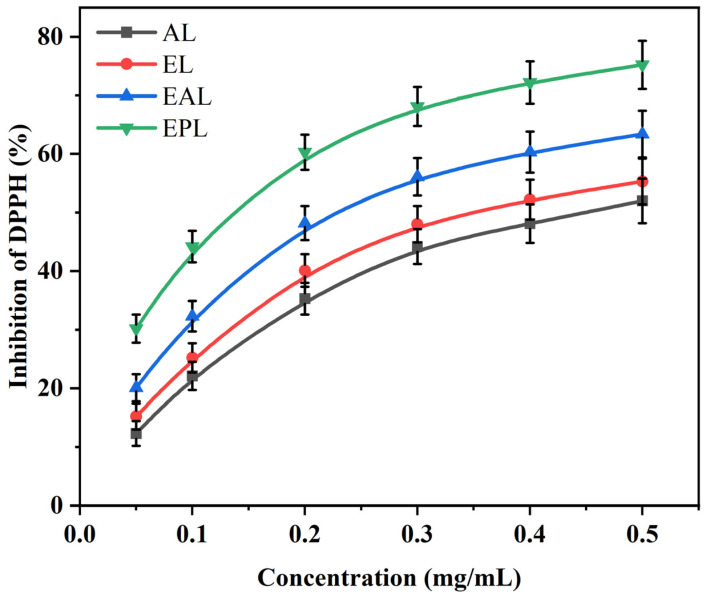
Antioxidant properties of alkali lignin (AL) and lignins isolated by different PIL pretreatments (EL, EAL, EPL).

The antioxidant activity of lignin samples obtained by different pretreatment methods was evaluated using the DPPH radical scavenging assay. DPPH is a stable organic radical with a characteristic absorption at 517 nm; the scavenging mechanism involves the transfer of a hydrogen atom or an electron from the antioxidant to form a stable non-radical DPPH-H complex. The DPPH scavenging curves of the five samples as a function of concentration are shown in Figure 7, and the corresponding half-maximal inhibitory concentration (IC_50_) values are listed in Table 4.

As shown in Figure 7, all lignin samples scavenged DPPH radicals, and the scavenging activity gradually increased with concentration from 0.05 mg/mL to 0.5 mg/mL. Among them, EAP lignin (EPL) and the positive control BHT exhibited higher scavenging activity within the tested concentration range, whereas AL showed the lowest activity. For example, at a concentration of 0.5 mg/mL, the scavenging activity of EPL was 75.2 ± 4.1%, which was higher than those of EL (55.3 ± 3.9%) and AL (52.0 ± 3.8%). This order was positively correlated with the total phenolic hydroxyl content of each lignin sample, indicating that phenolic hydroxyl groups are a key structural factor influencing antioxidant activity.

The IC_50_ value reflects the concentration required to scavenge 50% of DPPH radicals; a lower value indicates stronger antioxidant activity. As shown in Table 4, the IC_50_ of EPL was 0.17 ± 0.02 mg/mL, which was lower than those of EL (0.39 ± 0.03 mg/mL), EAL (0.27 ± 0.02 mg/mL), and AL (0.46 ± 0.04 mg/mL). These results indicate that EAP pretreatment, while achieving lignin separation, enhanced the antioxidant activity of lignin by increasing the total phenolic hydroxyl content to 2.26 ± 0.08 mmol/g and reducing its molecular weight. This observation is consistent with the increase in phenolic hydroxyl groups and the decrease in S/G ratio observed by ^31^P NMR and 2D-HSQC NMR analyses.

From a structure–activity perspective, the enhanced antioxidant activity of EPL is related to its high phenolic hydroxyl content (2.26 ± 0.08 mmol/g), low molecular weight (approximately 1800 g/mol), and low S/G ratio (0.71 ± 0.03). In particular, the enrichment of G-type phenolic hydroxyl groups (1.51 mmol/g) further improved the radical scavenging efficiency. The IC_50_ value of EPL is comparable to that reported by Gao et al. for PIL-treated technical lignin (0.15 mg/mL). Song et al. found that lignin extracted by deep eutectic solvents exhibited good thermal stability, UV shielding performance, and antioxidant activity, suggesting potential applications as a natural antioxidant substitute and additive in functional materials. These results confirm that EAP pretreatment, by enabling simultaneous lignin separation and structural activation under mild conditions, produces a lignin product that can serve as a natural antioxidant raw material.

## 4. Conclusions

Under the conditions of 120 °C and 40 min, the ethanolamine pyruvate (EAP) pretreatment of eucalyptus achieved a lignin separation yield of 79.0 ± 0.6%, with dissolution yields of cellulose and hemicellulose of 9.6 ± 0.3% and 11.2 ± 0.4%, respectively. The purity of the isolated EAP lignin (EPL) was 98.4 ± 0.3%. Compared with AL, the relative content of β-O-4 ether linkages in EPL decreased from 18.4 ± 0.4% to 14.2 ± 0.3%, the total phenolic hydroxyl content was 2.26 ± 0.08 mmol/g, the S/G ratio declined from 1.72 ± 0.04 to 0.71 ± 0.03, and the molecular weight was reduced. These data indicate that EAP pretreatment enables simultaneous selective lignin separation and structural modification (reduction of ether linkages, increase in phenolic hydroxyl groups, and decrease in S/G ratio). Among ethanolamine-based protic ionic liquid systems, EAP exhibits a favorable combination of separation selectivity and structural tunability. It should be noted that this study primarily observes lignin structural changes using characterization techniques such as NMR and GPC; confirmation of the specific roles of each component in the EAP system awaits further studies using model compounds. The DPPH radical scavenging assay showed that the antioxidant activity of EPL (IC_50_ = 0.17 ± 0.02 mg/mL) was higher than those of EL (0.39 ± 0.03 mg/mL) and AL (0.46 ± 0.04 mg/mL).

## Figures and Tables

**Table 1:** Composition of raw eucalyptus and pretreated residues after different pretreatment methods.

Sample	Solid Recovery Yield (%)	Cellulose Separation Yield (%)	Hemicellulose Separation Yield (%)	Lignin Separation Yield (%)	Ash Content (%)
Raw eucalyptus	—	46.8 ± 0.5	19.6 ± 0.4	25.3 ± 0.4	3.4 ± 0.1
ETA pretreatment	42.3	11.3 ± 0.4	38.7 ± 0.5	76.2 ± 0.6	2.1 ± 0.1
ETA/AcOH pretreatment	43.9	10.9 ± 0.4	33.2 ± 0.5	72.4 ± 0.6	2.0 ± 0.1
EAP pretreatment	46.5	9.6 ± 0.3	11.2 ± 0.4	79.0 ± 0.5	2.3 ± 0.1

**Table 2:** Key performance indicators of different lignin samples (mean ± SD, *n* = 3).

Indicator	Ethanolamine Lignin (EL)	Ethanolamine Acetate Lignin (EAL)	EAP Lignin (EPL)
Lignin separation yield (%)	76.2 ± 0.6	72.4 ± 0.6	79.0 ± 0.5
Purity (%)	94.7 ± 0.3	97.0 ± 0.3	98.4 ± 0.3
Total phenolic hydroxyl content (mmol/g)	1.41 ± 0.06	1.67 ± 0.07	2.26 ± 0.08
Mw (g/mol)	8736 ± 350	5836 ± 230	5519 ± 220
Mn (g/mol)	6723 ± 270	4524 ± 180	4415 ± 180
S/G ratio	1.08 ± 0.03	0.78 ± 0.02	0.71 ± 0.03

**Table 3:** Representative examples of EAP and other ethanolamine-based PIL systems.

Ionic Liquid System	Feedstock	Pretreatment Conditions	Lignin Separation Yield	Key Results	Reference
[MEA][OAc] (monoethanolamine acetate), ABR = 0.1 (amine excess)	Sugarcane bagasse	150 °C, 2 h	84%	Lignin extraction yield increased with higher base content, reaching 84% at ABR = 0.1; glucose release rate 96% (72 h enzymatic hydrolysis); solvent recovery up to 97% (ABR = 1.0)	Nakasu et al. [20]
HAc-[EOA][OAc] (acetic acid catalysis + ethanolamine acetate)	Poplar wood	First HAc catalysis at 170 °C for 0.5 h, then [EOA][OAc] pretreatment at 140 °C for 3 h	≈46%	Removed ≈ 88% of hemicellulose and extracted ≈ 46% of lignin; enzymatic glucose yield > 80%	Huang et al. [29]
[EOA][HOAc] (ethanolamine acetate)	Technical lignin	105 °C, 2 h	—	Phenolic hydroxyl content increased from 0.95 to 2.20 mmol/g (or by 215.69%); methoxy removal rate 44.73%; IC_50_ decreased to 0.15 mg/mL	Zhao et al. [24]
[EOA][GA] (ethanolamine glutarate)	Technical lignin	110 °C, 1.5 h	—	Polyphenol content increased by a factor of 1.58; β-O-4 bond cleavage; S/G ratio decreased; ionic liquid recovery > 95%	Li et al. [54]
[2-HEA][OAc] (2-hydroxyethylammonium acetate)	Eucalyptus	150 °C, 6 h, 10 wt% biomass loading	34.6%	Achieved approximately 75% glucan digestibility and 34.6% lignin removal; observed growth of Rhodosporidium toruloides at IL concentrations ≤ 5 *w*/*w*%; S/G ratio of residual lignin decreased	Rigual et al. [55]
Ethanolamine pyruvate (EAP)	Eucalyptus	120 °C, 40 min	79.0 ± 0.5%	Separation yields: lignin 79.0 ± 0.5%, cellulose 9.6 ± 0.3%, hemicellulose 11.2 ± 0.4%; lignin purity 98.4 ± 0.3%; phenolic hydroxyl content 2.26 ± 0.08 mmol/g; S/G ratio decreased from 1.72 ± 0.05 to 0.71 ± 0.03	This work

**Table 4:** IC_50_ values of alkali lignin (AL) and lignins isolated by different PIL pretreatments (EL, EAL, EPL).

Sample	AL	EL	EAL	EPL
IC_50_ (mg/mL)	0.46 ± 0.04	0.39 ± 0.03	0.27 ± 0.02	0.17 ± 0.02

## Data Availability

The original contributions presented in this study are included in the article/Supplementary Materials. Further inquiries can be directed to the corresponding author.

## Supplementary Materials

The following supporting information can be downloaded at https://www.mdpi.com/article/10.3390/polym18091109/s1: Figure S1: FTIR of eucalyptus before and after treatment; Table S1: Chemical compositions of poplar wood raw materials, Table S2: Molecular weight of alkali lignin and separated lignin from eucalyptus, Table S3: Signal attribution of characteristic peaks in 31P NMR atlas, Table S4: Assign-ment of different structural signals in 2D-HSQC NMR, Table S5: Semi-quantitative analysis of alka-li lignin and separated lignin of eucalyptus, Table S6: Recycling performance of the EAP solvent.
