# Supplementary material for: Efficient Separation and Targeted Activation of Lignin by Ethanolamine Pyruvate Protic Ionic Liquid
Source: Polymers (Basel). 2026 Apr 30;18(9):1109. doi: 10.3390/polym18091109 (PMC13165695; doi:10.3390/polym18091109)
Supplement: Supplementary file 1 [file polymers-18-01109-s001.zip › polymers-4234835-supplementary.pdf]

Supplementary information

Efficient Separation and Targeted Activation of Lignin by Ethanolamine Pyruvate Protic Ionic Liquid

Liuli Zhu [1], Jiatian Zhu [1], Jingpeng Zhou [2], Qin Feng [1], Baojie Liu [1], Chengrong Qin [1], Chen Liang [1], Caoxing Huang [3] and Shuangquan Yao [1,*]

[1] Guangxi Key Laboratory of Clean Pulp & Papermaking and Pollution Control, School of Light Industrial and Food Engineering, Guangxi University, Nanning 530004, China;

[2] Shandong Huatai Paper Co., Ltd., Dongying & Shandong Key Laboratory of Biobased Material and Green Pulp Papermaking, Dongying 257335, China;

[3] Jiangsu Co-Innovation Center of Efficient Processing and Utilization of Forest Resources, Nanjing Forestry University, Nanjing 210037, China;

[*] Correspondence: yaoshuangquan@gxu.edu.cn

Supplementary information: 20 pages, 7 methods, 1 picture, 6 tables.

List of Methods:

Method 1. Calculation of separation yields

Method 2. Calculation of crystallinity index

Method 3. Calculation of surface lignin coverage

Method 4. 2D HSQC NMR analysis and semi-quantification of lignin structural units

Method 5. Gel permeation chromatography for molecular weight determination

Method 6. Purity and elemental analysis

Method 7. Quantification of hydroxyl groups by ^{31}P NMR

List of Picture:

Figure S1 FTIR of eucalyptus before and after treatment.

List of Tables:

Table S1 Chemical compositions of poplar wood raw materials.

Table S2 Molecular weight of alkali lignin and separated lignin from eucalyptus.

Table S3 Signal attribution of characteristic peaks in ^{31}P NMR atlas.

Table S4 Assignment of different structural signals in 2D-HSQC NMR.

Table S5 Semi-quantitative analysis of alkali lignin and separated lignin of eucalyptus.

Table S6. Recycling performance of the EAP solvent.

Method

1. Calculation of separation yield

The separation yield was calculated based on the change in chemical composition before and after pretreatment. First, 0.2 g (oven-dry weight) of wood powder (40–60 mesh) was weighed into a 50 mL iodine flask. Then, 9 mL of 72% (w/w) sulfuric acid was added, and the mixture was shaken at 120 rpm in a water bath at 30 °C for 1 h. After this initial hydrolysis, the acid solution was diluted to 4% (w/w) by adding 252 mL of ultrapure water in a glass bottle. The bottle was sealed and placed in an autoclave at 121 °C for 1 h. After cooling to room temperature, the solid residue was separated by filtration through a pre-weighed G4 sintered glass crucible (pore size 10–16 μm). The residue was washed with ultrapure water until the filtrate became neutral (pH $\approx$ 7) and then dried in an oven at 105 °C to constant weight. The acid-insoluble lignin content was calculated using Equation S1.

For acid-soluble lignin, an aliquot of the combined filtrate was diluted appropriately with 4% H_2SO_4, and the absorbance at 205 nm was measured using a UV-Vis spectrophotometer (Shimadzu UV-2600, Tokyo, Japan). The acid-soluble lignin content was calculated using Equation S2, with an absorptivity of 110 $L\cdot g^{-1}\cdot cm^{-1}$. Total lignin content is the sum of acid-insoluble and acid-soluble lignin (Equation S3).

For monosaccharide analysis, a small portion of the filtrate was centrifuged at 10,000 rpm for 10 min, and the supernatant was filtered through a 0.22 μm nylon membrane. The concentrations of glucose and xylose were determined by

high-performance liquid chromatography (HPLC, Alliance E269, Waters, Massachusetts, USA). Cellulose and hemicellulose contents were then calculated using Equations S4 and S5, applying the typical anhydro correction factors. The calculation formula of each lignin content is as follows:

Equation S1: $m_{iL}=\frac{m_{SR}}{W}\times100\%$

Equation S2: $m_{sL}=D\times A\times\frac{V}{100\times W}\times100\%$

Equation S3: $m_L=m_{iL}+m_{sL}$

where: m_{iL} is acid-insoluble lignin content, %; m_{iL} is acid-soluble lignin content, %;m_L is the total lignin content, %; m_{SR} is the residue mass, g; W is the absolute dry mass of wood flour, g; D is the dilution ratio; A is the light absorption value; V is the filtrate volume, L.

A small aliquot of the filtrate was centrifuged, and the resulting supernatant was passed through a 0.22 μm microporous membrane filter. The concentrations of five monosaccharides in the acid hydrolysate were determined by HPLC. The cellulose and hemicellulose contents were calculated accordingly. The formula is as follows:

Equation S4: $m_C=D\times C_G\times\frac{V}{W}\times0.90\times100\%$

Equation S5: $m_H=D\times C_X\times\frac{V}{W}\times0.88\times100\%$

where: m_C is cellulose content, %; C_G is the glucose concentration, g/L; m_H is hemicellulose content, %; C_X is xylose concentration, %.

The separation yield is calculated by the content of each component in the raw material and the remaining solid, and the calculation formula is as follows:

Equation S6: $SR=\frac{M_1}{M_0}\times 100\%$

Equation S7: $SY_C=\frac{m_{C0}\text{-}SR\times m_{C1}}{m_{C0}}\times 100\%$

Equation S8: $SY_H=\frac{m_{H0}\text{-}SR\times m_{H1}}{m_{H0}}\times 100\%$

Equation S9: $SY_L=\frac{m_{L0}\text{-}SR\times m_{L1}}{m_{L0}}\times 100\%$

where: SR is solid recovery rate, %; M_0 is the absolute dry mass of raw materials, g; M_1 is the absolute dry mass of different pretreatment samples, g; m_{ij} is the content of three components (C: cellulose, H: hemicellulose, L: lignin, 0: untreated sample, 1: treated sample),%; SY_i is the separation yield of three components (C: cellulose, H: hemicellulose, L: lignin),%.

$$\text{Lignin separation yield (\%)} = \frac{\text{mass of lignin recovered from the pretreatment liquor}}{\text{mass of lignin in the raw eucalyptus}} \times 100\%$$

2. Calculation of crystallinity index

Crystallinity index (CrI) was determined using X-ray diffraction (XRD) on a Rigaku SmartLab 9 kW diffractometer (Rigaku, Tokyo, Japan). The CrI was calculated based on the peak height method (Kurakake et al., 1994) using Equation S10:

Equation S10: $CrI=(I_{002}\text{-}I_{am})/I_{002}\times 100\%$

where: I_{002} is the diffraction peak intensity of about 2θ=22.5° in the crystalline region, I_{am} is the diffraction peak intensity of the amorphous part of the sample about 2θ=18°.

3. Calculation of surface lignin coverage

Surface lignin coverage (SLC) was calculated from the oxygen-to-carbon (O/C) atomic ratios obtained by X-ray photoelectron spectroscopy (XPS). The surface lignin

coverage was calculated according to Chu et al. (Chu et al., 2021) using Equation S11:

Equation S11:$SRL = (\frac{O}{C_S} - \frac{O}{C_C})/(\frac{O}{C_S} - \frac{O}{C_1})$

where: $\frac{O}{C_S}$ is the O/C value of the sample, $\frac{O}{C_C}$ is the O/C value of cellulose (0.83), $\frac{O}{C_1}$ is the O/C value of lignin (0.33)

4. 2D HSQC NMR analysis and semi-quantification of lignin structural units

The 2D HSQC NMR spectra of the lignin samples were acquired using a nuclear magnetic resonance spectrometer. The acquisition temperature was 25 °C, and deuterated dimethyl sulfoxide (DMSO-d_6) was used as the solvent. Approximately 80 mg of each lignin sample was weighed into a 2 mL vial, followed by the addition of 700 μL of DMSO-d_6. The mixture was vortexed to dissolve the lignin and then ultrasonicated to remove air bubbles. The resulting solution was homogeneous and free of visible particles. The fully dissolved solution was transferred into an NMR tube for measurement. The pulse sequence used was hsqcetgpsp.3 (or hsqcetgpsi). The number of data points (TD) was 2048 in the F2 dimension and 512 in the F1 dimension. The acquisition time (AQ) was 0.178176 s, with 64 scans (NS) and a relaxation delay (D1) of 1.5 s (or 0.98976 s). The spectral width (SW) was 11.4912 ppm (δH) in the F2 dimension and 120 ppm (δC) in the F1 dimension. The center frequencies (O1P) were 3.176 ppm in the F2 dimension and 100 ppm in the F1 dimension. Data processing was performed using Bruker-Topspin 2.1 software. Volume integrals were normalized against the total signal intensity of the aromatic region to calculate the relative content of each structural unit. The S/G ratio was calculated from the integral intensity ratio of

the $S_{2,6}$ and G_2 signals. The relative content of each inter-unit linkage (per 100 aromatic units) was calculated from the ratio of the integral intensity of the corresponding α-site signal to the total integral intensity of the aromatic ring. Semi-quantitative analysis of lignin was performed using the 2D HSQC method according to the following equations:

Equation S13: $IC_9 = 0.5IS_{2,6} + IG_2 + 0.5IS'_{2,6}$

Equation S14: $\frac{S}{G} = \frac{0.5(S_{2,6}+S'_{2,6})}{IC_9}$

Equation S15: $\beta - O - 4 = \frac{A_\alpha(S)}{IC_9}$

Equation S16: $\beta - \beta = \frac{B_\alpha}{IC_9}$

Equation S17: $\beta - 5 = \frac{C_\alpha}{IC_9}$

where $IS_{2,6}$ and $IS'_{2,6}$ are the integrals of $S_{2,6}$ and $S'_{2,6}$, respectively; IG_2 is the integral of G_2; I_A is the integral of β-O-4; I_B is the integral of β-β; and I_C is the integral of β-5. Each sample was measured in triplicate, and results are expressed as mean ± standard deviation.

5. Gel permeation chromatography (GPC) for molecular weight determination

The weight-average molecular weight (Mw) and number-average molecular weight (Mn) of lignin were determined by gel permeation chromatography (GPC, PL-GPC50, Waters, USA). Two different mobile phase systems were used depending on the solubility of the lignin samples. For water-soluble lignin (e.g., lignin extracted by EAP), an aqueous mobile phase system was employed. Sample preparation: accurately weigh 5 mg of lignin, dissolve in 1 mL of 0.1 M NaOH solution, filter through a 0.22 μm aqueous membrane filter, and then inject. For milled wood lignin (MWL), an organic

phase system was used. Sample preparation: accurately weigh 5 mg of lignin, dissolve in 1 mL of THF, filter through a 0.22 μm organic membrane filter, and then inject.

Calibration curves were constructed using narrow-distribution standards. For the aqueous system, poly (sodium styrenesulfonate) standards with molecular weights ranging from 1100 to 100,000 Da were used. For the organic system, polystyrene standards with molecular weights ranging from 580 to 316,000 Da were used. The standard curves were established based on the relationship between retention time and the logarithm of molecular weight, from which the Mw and Mn of the samples were calculated. The polydispersity index (PDI) was calculated as Mw/Mn. Each sample was measured in triplicate, and results are expressed as mean ± standard deviation.

6. Purity and Elemental analysis

Lignin purity was determined using a one-step method. A 5 mg sample was placed in a 5 mL headspace vial, followed by the addition of 1.475 mL of 9.5 wt% H_2SO_4. The vial was then sealed with a dedicated headspace cap. The reaction was carried out in an oven at 105 °C for 2.0 h. To ensure complete reaction, the vial was shaken every 15 min. After the reaction, the vial was immediately cooled to terminate the process. The supernatant from the cooled reaction mixture was diluted 20-fold. Specifically, 5 mL of distilled water was first added to a 10 mL volumetric flask, then 500 μL of the supernatant was transferred into the flask using a micropipette, and finally distilled water was added to the mark. The diluted solution was filtered through a 0.22 μm aqueous syringe filter (polyethersulfone, PES). The absorbance of acid-soluble lignin

was measured at 205 nm using a UV-Vis spectrophotometer. The mass of acid-soluble lignin (B) was calculated according to Equation S18.

Acid-insoluble lignin was determined gravimetrically. A G4 sintered glass crucible was first dried to constant weight and weighed (m_1). After acid hydrolysis, the sample was filtered through the pre-weighed crucible. The crucible with the residue was dried in an oven at 105 °C for 6 h, cooled to constant weight, and weighed (m_2). The mass of acid-insoluble lignin was obtained as m_2–m_1. The total lignin mass was the sum of acid-soluble and acid-insoluble lignin. Lignin purity was then calculated as the total lignin mass divided by 5 mg.

Equation S18: $B = \frac{A}{110} \times D \times 1.475$

where: B is mass of acid-soluble lignin in the 5 mg sample (mg); A is absorbance at 205 nm; 110 is absorptivity coefficient ($L \cdot g^{-1} \cdot cm^{-1}$); D is dilution factor (here, D=20).

Elemental compositions (C, H, N, S) of lignin samples were analyzed using an Elementar Vario EL cube elemental analyzer (Elementar, Langenselbold, Germany). Approximately 2–3 mg of oven-dried sample was weighed into a tin capsule and combusted in a quartz tube at 1150 °C. The reduction tube was maintained at 850 °C. High-purity helium (99.999%) was used as the carrier gas at a flow rate of 200 mL/min. Oxygen (99.999%) was injected as a combustion aid (30 mL per sample). The resulting gases (CO_2, H_2O, N_2, SO_2) were separated on a GC column and detected by a thermal conductivity detector (TCD). The oxygen content was calculated by difference: 100%−(C%+H%+N%+S%+ash%).

Ash content was determined following the NREL standard method NREL/TP-510-42622. An oven-dried sample (2 g) was accurately weighed into a pre-weighed ceramic crucible, placed in a muffle furnace, and heated at a rate of 5–10 °C/min to 575 ± 25 °C. The temperature was maintained for 4 h. After incineration, the furnace was allowed to cool to approximately 200 °C. The crucible was then transferred to a desiccator and cooled to room temperature for about 1 h before weighing. Ash content was calculated using the following formula:

Equation S19: $Ash(\%) = \frac{m_{ash} - m_{sieve}}{m_{sample}} \times 100\%$

where m_{ash} is the mass of the crucible plus residue after incineration (g), m_{sieve} is the mass of the empty crucible (g), and m_{sample} is the mass of the oven-dried sample (g). Three independent replicate determinations were performed for each sample, and results are expressed as mean ± standard deviation.

7. Quantification of hydroxyl groups by ^{31}P NMR

The contents of different hydroxyl groups (aliphatic OH, phenolic OH, and carboxyl OH) in lignin were determined by ^{31}P NMR (Avance NEO 600, Bruker, Germany). An internal standard solution was first prepared: chromium (III) acetylacetonate ($Cr(acac)_3$) was used as the relaxation reagent and N-hydroxysuccinimide (NHS) as the internal standard. Both were dissolved in anhydrous pyridine to obtain a solution containing 50 mmol/L $Cr(acac)_3$ and 50 mmol/L NHS. For sample derivatization, 20 mg of dried lignin was accurately weighed into a 5 mL amber vial, followed by the addition of 400 μL of deuterated chloroform ($CDCl_3$)

and 200 μL of the internal standard solution. The mixture was vortexed until the lignin was completely dissolved. Then, 80 μL of 2-chloro-4,4,5,5-tetramethyl-1,3,2-dioxaphospholane (Cl-TMDP) derivatization reagent was added, and the vial was shaken continuously to ensure sufficient reaction between the hydroxyl groups of lignin and Cl-TMDP. After the reaction, the mixed solution was quickly transferred into a 5 mm NMR tube for measurement.

NMR spectra were acquired on a Bruker Ascend-500 MHz or Avance NEO 600 spectrometer under the following conditions: acquisition temperature 25 °C, pulse sequence zgig, number of scans 128, relaxation delay 6 s, pulse width 12 μs (90°), and acquisition time 1.5 s. Chemical shifts were referenced to the signal of the reaction product between Cl-TMDP and N-hydroxysuccinimide (δ 152.0 ppm) or to the signal of derivatized water (δ 132.2 ppm).

The content of each hydroxyl type (mmol/g) was calculated according to the following formula:

Equation S20: $$Hydroxyl\ content\ (mmol \cdot g^{-1}) = \frac{I_{sample} \times n_{std} \times m_{std}}{I_{std} \times m_{sample} \times M_{std}}$$

where I_{sample} is the integrated area of the target hydroxyl signal of the sample, I_{std} is the integrated area of the internal standard signal, m_{std} is the number of hydroxyl groups per internal standard molecule (1 for N-hydroxysuccinimide), n_{std} is the mass of the internal standard added (mg), m_{sample} is the mass of the sample (mg), and M_{std} is the molar mass of the internal standard (115.09 g/mol). Each sample was measured in triplicate, and results are expressed as mean ± standard deviation.

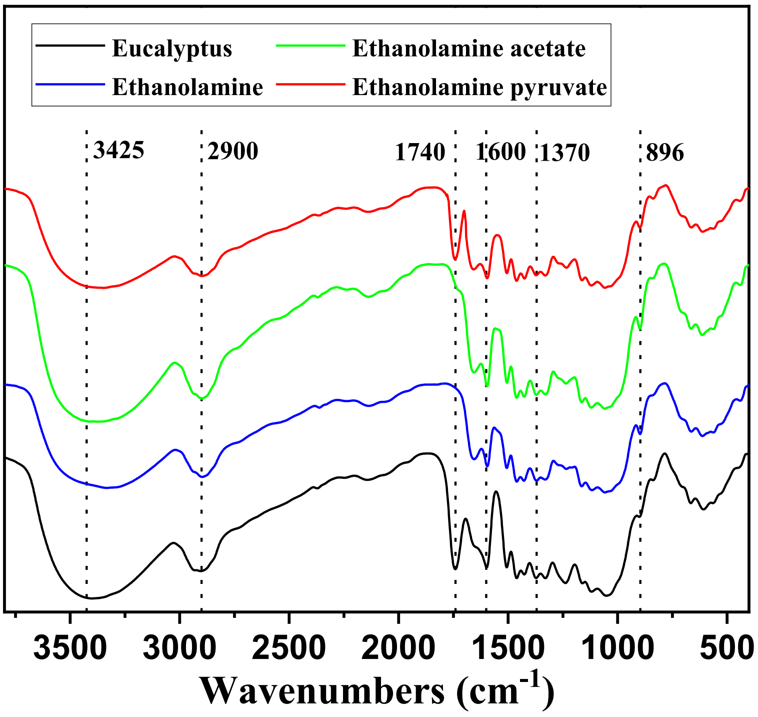

Figure S1. FTIR spectra of eucalyptus before and after treatment

(FTIR spectra of eucalyptus before (Raw) and after treatment with different ionic liquids (EL, EAL, EPL). Spectra were recorded on a Nicolet iS50 FTIR spectrometer (Thermo Fisher Scientific, USA) using the KBr pellet method (sample/KBr = 1:100, w/w) in the range of 4000–400 cm^{-1} with a resolution of 4 cm^{-1} and 32 scans.)

Table S1 Chemical composition of the raw material.

Sample	Cellulose (%)	Hemicellulose (%)	Lignin (%)	Benzene alcohol extract (%)
RM	46.8±0.5	19.6±0.4	25.3±0.4	1.6±0.1

Table S2 Table S2. Molecular weight of alkali lignin and isolated lignins from eucalyptus.

Sample	Mw (g/mol)	Mn (g/mol)	PDI
Alkali lignin (AL)	12700±500	8736±350	1.45±0.05
Ethanolamine lignin (EL)	8736±350	6723±270	1.30±0.04
Ethanolamine acetate lignin (EAL)	5836±230	4524±180	1.29±0.04
Ethanolamine pyruvate lignin (EPL)	5519±220	4415±180	1.25±0.04

Note: Molecular weights were determined by GPC as described in Method 5.

Table S3. Signal assignment of characteristic peaks in ^{31}P NMR spectra

Chemical shift (ppm)	Signal assignment
134.34 – 136.25	Carboxylic acid OH
138.87 – 141.14	G-type phenolic OH
142.03 – 144.21	S-type phenolic OH
145.53 – 150.12	Aliphatic OH
151.28 – 152.48	Internal standard

Note: Chemical shifts were obtained from ^{31}P NMR spectra acquired according to Method 7.

Table S4. Assignment of structural signals in 2D-HSQC NMR spectra

Label	δ_c/δ_H (ppm)	Assignment
B_β	53.0/3.46	C_β-H_β (B)in Resinol Structure
C_β	53.4/3.06	C_β-H_β (C) in phenylcoumarane
$-OCH_3$	55.6/3.73	C-H in methoxy group
A_γ	59.5/3.65	C_γ-H_γ (A') in the β-O-4 structure
C_γ	62.6/3.72	C_γ-H_γ (C) in phenylcoumarane
B_γ	71.2/3.81	C_γ-H_γ (B) in resinol structure
$(A, A')_\alpha$	71.7/4.81	C_α-H_α (A) in β-O-4 and γ-acylated β-O-4 (A')
B_α	84.8/4.63	C_α-H_α (B) in resinol structure
$A_{\beta(G/S)}$	84.0/4.28	C_β-H_β (A) in the β-O-4 bond of S-type lignin structural unit
$(A')_{\beta(S)}$	85.7/4.09	C_β-H_β(A') in the β-O-4' bond of S-type lignin structural unit
C_α	86.9/5.41	C_α-H_α (C) in phenylcoumarane
$S_{2,6}$	104.4/6.53	$C_{2,6}$-$H_{2,6}$ (S) in syringyl
G_2	110.8/6.91	C_2-H_2 (G) in guaiacyl
G_5	114.7/6.67	C_5-H_5 (G) in guaiacyl
G_6	119.2/6.70	C_6-H_6 (G) in guaiacyl
$Pb_{(2,6)}$	131.5/7.73	C_2-H_2 and C_6-H_6 in p-benzoic acid (Pb)

Note: Signal assignments were based on 2D-HSQC NMR spectra acquired as described in Method 4.

Table S5. Semi-quantitative analysis of alkali lignin and isolated lignins from eucalyptus

Structure	Content (%)			
	AL	EL	EAL	EPL
β-O-4	18.42	17.34	16.25	14.19
β-5	2.61	2.57	2.54	2.64
β-β	2.93	2.87	3.01	2.94
S	63.27	51.97	43.68	41.83
G	36.73	48.03	56.32	58.17

Note: Semi-quantification was performed using 2D-HSQC NMR as described in Method 4.

Table S6 Recycling performance of the EAP solvent: separation yields of cellulose, hemicellulose, and lignin at different cycle numbers (1–5), and solvent recovery rate.

Cycle time	DES recovery (%)	Lignin (%)	Cellulose (%)	Hemicellulose (%)
1	94.5 ± 0.4	79.0 ± 0.4	9.6 ± 0.3	11.2 ± 0.3
2	91.4 ± 0.5	78.2 ± 0.4	9.9 ± 0.4	11.6 ± 0.3
3	87.7 ± 0.5	77.4 ± 0.5	10.4 ± 0.4	12.1 ± 0.4
4	83.2 ± 0.6	76.6 ± 0.6	10.9 ± 0.4	12.7 ± 0.5
5	79.8 ± 0.7	75.6 ± 0.6	11.5 ± 0.5	13.4 ± 0.5
